# Systematic Survey of the Alteration of the Faecal Microbiota in Rats With Gastrointestinal Disorder and Modulation by Multicomponent Drugs

**DOI:** 10.3389/fphar.2021.670335

**Published:** 2021-11-02

**Authors:** Yue Wu, Yang Wu, Hongwei Wu, Changxun Wu, Enhui Ji, Jing Xu, Yi Zhang, Junying Wei, Yi Zhao, Hongjun Yang

**Affiliations:** ^1^ Institute of Chinese Materia Medica, China Academy of Chinese Medical Sciences, Beijing, China; ^2^ Medical Experimental Center of Chinese Academy of Traditional Chinese Medicine, Beijing, China; ^3^ Key Laboratory of Intelligent Information Processing, Advanced Computer Research Center, Institute of Computing Technology, Chinese Academy of Sciences, Beijing, China

**Keywords:** XEFP, functional dyspepsia, intestinal flora, metabonomics, network pharmacology

## Abstract

Gastrointestinal disorder (GID) is a global health disease which leads to heavy public medical burden. Disorders in the intestinal flora have been found in gastrointestinal disorder patients. However, the interaction between GID and the intestinal flora in faecal has not been studied comprehensively. In addition, multicomponent drugs represented by traditional Chinese medicine (TCM) are widely used for treating GID, but their modulation of the intestinal flora has not been investigated. Therefore, in this study, a high-throughput sequencing strategy was used to investigate alterations in the intestinal flora in a rat GID model, followed by an investigation of the modulation by a representative TCM, Xiaoerfupi (XEFP) granule. The results showed that in rats with GID, the relative abundances of Erysipelotrichaceae, Lachnospiraceae, Streptococcaceae increased and that of Ruminococcaceae decreased. At the macro level, the levels of LysoPC(16:0), LysoPC(20:2), LysoPC(15:0), LysoPC(20:2 (11Z, 14Z)), LysoPC(20:1), LysoPC(15:0), LysoPC(20:0) and LysoPE (0:0/20:0) in serum increased and levels of PC(36:4), PC(38:4), PC(o-36;4), PE (MonoMe(13,5)/MonoMe(11,5)) decreased. The imbalance of metabolites was restored by XEFP through *ether lipid metabolism* pathway. Increase in the phyla Firmicutes/Bacteroidetes (F/B) ratio of the GID rats was restored by XEFP as well. Moreover, XEFP can relief the symptoms of GID rats by increasing bacteria Ruminococcaceae and decreasing Streptococcaceae*,* Erysipelotrichaceae and Lachnospiraceae in faecal microbiota level. This study represents a comprehensive survey of the interaction between GID and the intestinal flora and a systematic evaluation of modulation by a multicomponent drug.

## Introduction

Gastrointestinal disorder (GID), including functional dyspepsia (FD), gastroparesis (GP) and others, is a global health disease which leads to heavy public medical burden. Chronic gastric hypersensitivity and gastric motor dysfunction can be observed in GID, which makes patients suffer from severe pain or burning along with postprandial fullness, that greatly reduces their quality of life (Kim and Kim, 2020). Many factors contribute to the prevalence of GID, including disturbances in gastric motor function, gastrointestinal infections and other pathological states (Pike et al., 2013), as well as some lifestyle factors, such as overweight and obesity (Jung et al., 2016) and anxiety and depression (Deding et al., 2017). Considering the high morbidity and paucity of effective treatment strategies for GID, the treatment situation is unsatisfactory (Selgrad and Malfertheiner, 2008).

Trillions of microbes constitute the intestinal flora, which, when disequilibrated, cause a human microecological imbalance (De Vadder et al., 2014; Forsythe et al., 2016). The intestinal flora modulate not only the peripheral but also the central nervous system (CNS) (Naveed et al., 2021). Disorders of the gut-brain interaction, including irritable bowel syndrome (IBS), functional GID, and abdominal migraine, are often related to disorders of the intestinal flora (Thapar et al., 2020). It has been reported that development of IBS, a functional gastrointestinal disorder, is related to the consumption of particular foods, and it can be assumed that the gastrointestinal microbiota has its own specific way of influencing the pathological state (Böhn et al., 2013).

In patients with GID, disorder of the intestinal flora is always present and is considered to be related to GID morbidity. Alterations in the gastrointestinal microbiota, particularly certain proinflammatory bacteria, in patients with GID, have a synergistic effect on the inflammation of the small intestine, which results in an exacerbation of GID in patients (Pavlidis et al., 2015; Talley and Ford, 2015). Additionally, changes in the intestinal flora can contribute to altering human metabolites *via* specific metabolic pathways, particularly those influencing short chain fat acids (SCFAs) (Koduru et al., 2018; Ford et al., 2020). Furthermore, disturbances in the gastrointestinal microbiome can influence the gut-brain axis *via* hormone pathways (Rodiño-Janeiro et al., 2015). Although the microbiota is considered a potential factor for GID, the difficulty in interpreting the relative bacterial abundance makes it difficult to identify correlations between microbiota and GID (Zhong et al., 2017). Further studies are needed to examine the interaction between GID and the intestinal flora.

Multicomponent drugs represented by traditional Chinese medicine (TCM) are widely used for treating GID. In some clinical trials in patients with GID, TCM has demonstrated efficacy (Wu et al., 2011; Chen et al., 2020; Xu et al., 2020). Xiaoerfupi (XEFP) granule is a TCM that is composed of six medicinal plant-derived natural products (Ji et al., 2019), including *Atractylodes macrocephala*, *Pericarpium citri reticulatae*, *Poria coss*, *Codonopsis pilosula*, *Crataegus pinnatida*, and *Nelumbo nuclefera*. Due to its favourable clinical efficacy and accessibility, XEFP has become a common aqueous decoction for treating GID and alleviating GID symptoms in China. In a previous work, we performed a pharmacodynamic evaluation (Ji et al., 2019) of XEFP and predicted its potential targets (Wei et al., 2019), but whether XEFP influences the intestinal flora and the affected microbiota and corresponding mechanism have not yet been reported.

In this study, we detected alterations in the intestinal microbiota of faecal in rats with GID using a high-throughput sequencing strategy and revealed the relationship between the intestinal flora and GID. Then, the effect of XEFP on the regulation of the intestinal bacterial flora of rats with GID was investigated, followed by metabolomics-based verification. This study involved a comprehensive survey of the interaction between GID and the intestinal flora and systematically evaluated the modulation of this interaction by a multicomponent drug.

## Materials and Methods

### Materials

XEFP was provided by Hunan Timesun Pharmaceutical Co., Ltd (Yongzhou, China). Iodoacetamide (IAA) was purchased from Sigma-Aldrich (Beijing, China). Domperidone (DOM) was provided by Xi’an Janssen Pharmaceutical, Ltd (Xi’an, China). Pentobarbital sodium was purchased from Sigma (United States).

### Animal Study

A GID rat model was established using transient neonatal gastric irritation and alternate-day fasting (Liu et al., 2008). Sprague–Dawley rats (male; 10 days old; Vital River Laboratory Animal Technology Co., Ltd, Beijing, China) received 0.2 ml/100 g 0.1% IAA in 2% sucrose by oral gavage for 6 days, while the control group received 0.2 ml of 2% sucrose. Forty-two days later, rats treated with IAA were fed under alternate-day fasting for 14 days. Rats were then divided into six groups: model (received 5 ml/kg water per day by oral gavage, n = 10); domperidone (DOM) (DOM received 3 mg/kg per day by oral gavage, n = 10); XEFP-H (received XEFP 80 mg/kg per day by oral gavage, n = 10); XEFP-M (received XEFP 40 mg/kg per day by oral gavage [equivalent dose for humans], n = 10); XEFP-L (received XEFP 20 mg/kg per day by oral gavage, n = 10); control (sucrose-treated rats, n = 8). All rats were treated with 5 ml/kg each drug or water each day for 21 days. Excrement was collected into sterile tubules every morning during normal feeding. After rats were narcotized with 1% Pentobarbital sodium, blood samples were collected from the abdominal aortas. Serum was isolated at 3,240 r/min (15 min, 4°C), and the supernatant was stored at −20°C for further research. All animal care and experimental protocols were approved by the Committee on Animal Care and Use of the Institute of Chinese Materia Medica, China Academy of Chinese Medical Sciences.

### Pharmacodynamic Evaluation of XEFP in Rats With GID

After 12 h of fasting, rats were randomly selected to perform the gastric emptying test (6 rats in each group). The selected rats were housed in individual cages and supplied with enough food for 3 h (21 g/rat). After 3 h, the weight of food remaining in the cage was weighed as the food remaining (Wr). The selected rats were fasted, including water, for 3 h. Finally, the rats were narcotized with 1% Pentobarbital sodium and sacrificed, and the stomachs were removed, cleaned, and weighed as both the total weight (Wt) and suttle weight (Ws). The rate of gastric emptying was calculated as follows:

$$\text{Gastric}\;\text{emptying}\;\left(\%\right)=\left(1-\frac{Wt-Ws}{21-Wr}\right)\times 100\%$$

Serum levels of perinterleukin 4 (IL-4), interferon-γ (IFN-γ), amylase (AMS), lactic acid (LD), motilin (MTL), gastrin (GAS), somatostatin (SS), calcitonin gene-related protein (CGRP), nitric oxide synthase (NOS), and vasoactive intestinal peptide (VIP) were measured using enzyme-linked immunosorbent assay (ELISA) kits (Novus, Hong Kong). Each sample collected from the rats was centrifuged at 3,240 r/min (15 min, 4°C), and then, the supernatant was assayed according to the manufacturer’s instructions. A specific primary antibody against a target protein was immobilized on a 96-well plate. The target protein in sera was recognized by the first antibody and then incubated with a horseradish peroxidase-conjugated secondary antibody for 20 min at the proper temperature (37°C). Finally, the samples were exposed to a 450 nm illuminant, and the optical density was recorded. The levels of the cytokines we observed were determined according to a standard curve.

### Intestinal Flora Analysis in Rats With GID

Total DNA was extracted from the faecal samples we collected using the SDS-polyacrylamide gel electrophoresis (SDS-PAGE). DNA purification and concentration were performed by agarose gel electrophoresis, and DNA was diluted in sterile water to 1 ng/L. High-fidelity PCR Master Mix GC Buffer with specific barcode primers provided by New England Biolabs Specific was used for PCR with the diluted DNA as a template.

The following primers were used: the 16S V4 primers (515f and 806r) were used to identify bacterial diversity; 18S V4 primers (528f and 706r) were used to identify eukaryotic diversity; and ITS1 primers (its5-1737f and its2-2043r) were used to identify fungal diversity. Additional amplified regions included the 16S v3-v4/16S v4-v5; Archaea 16S V4; 18S V9; and ITS2 regions. A library was built using the TruSeq DNA PCR-Free Sample Preparation Kit. HiSeq2500 PE250 was used for sequencing after the library was qualified, and raw tags were spliced using FLASH (V1.2.7) (Magoc and Salzberg, 2011). The sequencing reads in FASTA format for all samples were submitted to the SILVAngs web server (https://www.arb-silva.de/ngs) with default parameters. In brief, all input reads were firstly aligned by SINA to remove problematic reads, possible contamination, or redundancy. And then the remaining reads were used to map the SILVA SSUrRNA database for classification of operational taxonomic units (OTUs). Then the OTU abundance spectrum and other statistical data were obtained. Functional spectral information was obtained by importing the results from SILVAngs to the R package Tax4Fun. Finally, functional profiles (KEGG) of the microbial communities were predicted. All analyses were based on the SILVA database.

To better understand the alterations in GID, we also uploaded the OTU abundance spectrum to MENA and performed Spearman correlation analysis on the OTUs present in at least 50% of the sample. The equation is shown below:

$$r_{s}=\rho_{rgX,rgy}=\frac{cov\left(rg_{X},rg_{Y}\right)}{\sigma_{rgX}\sigma_{rgY}}$$

The chosen threshold value (0.640) was calculated by random matrix theory to construct a network. The simulated annealing approach calculation method was used to zone the network to 44 modules, called CAGs. The modules were uploaded to Cytoscape. SPSS was used to perform a *t*-test on the summary of OTU relative abundance. Significant statistics were plotted using GraphPad Prism 8.

### Serum Metabolomic Analyses

100 μl serum sample mixed with 300 μl of cold acetonitrile was vortexed vigorously for 30 s then centrifuged at 4°C at 12,000 rpm for 10 min and the supernatant was injected into the UPLC-Q/TOF–MS analytical system.

An untargeted metabonomics approach based on complementary hydrophilic interaction chromatography (HILIC) and reversed-phase liquid chromatography (RPLC) combined with hybrid quadrupole-time-of-flight Q-TOF mass spectrometry was implemented. For HILIC-MS analysis, a Waters UPLC BEH amide column (2.1 mm ⅹ 100 mm, 1.7 µm particle size) was used for the separation. The condition was set as follow: 1. Mobile phase: contained solvent A (0.1% formic acid—acetonitrile, containing 1 mM ammonium formate) and solvent B (0.1% formic acid—water, containing 2 mM ammonium formate). 2. Elution method: Gradient elution (0–1 min, 95% A; 1–9 min, 95–50% A; 9.1–13 min, 50–95% A). 3. The flow rate of the mobile phase: 0.3 ml min-1.4. The column temperature: 40°C 5. The injection volume: 1 μl. For RPLC-MS analysis, A Waters ACQUITY HSS T3 (2.1 × 100 mm, 1.8 µm) system was used for the separation procedure. The condition was set as follow: 1. The mobile phase: consisted of solvent A (0.1% formic acid - water) and solvent B (0.1% formic acid - methanol). 2. Elution method: Gradient elution (0–1 min, 100% A; 1–4 min, 100–30% A; 4–12 min, 30–0% A; 12.1–14 min, 0–100% A). 3. The flow rate of the mobile phase: 0.3 ml min-1.4. The injection volume was 1 μl.

The conditions of MS analysis were as follows: 1. Both RPLC-MS and HILIC-MS spectra were acquired on a Q-TOF mass spectrometer (Xevo G2 Q-TOF MS systems, Waters Corp, Milford, MA, United States), equipped with an electrospray ionization (ESI) source. 2. Scan parameter: The full-scan data were acquired from 50 to 1,200 Da, along with a scan time of 0.2 s using a capillary voltage of 3.0 kV for positive mode and 2.2 kV for negative mode, a desolvation temperature of 350°C, sample cone voltage of 40 V, extraction cone voltage of 4 V, source temperature of 100°C, cone gas flow of 40 L/h and desolvation gas flow of 800 L/h. The mass spectrometer was calibrated across a mass range of 50–1,200 Da using a solution of sodium formate. The mass was corrected during acquisition using an external reference (Lock-Spray™) consisting of a 0.2 ng ml-1 solution of leucine enkephalin, infused at a flow rate of 5 μl min-1 *via* a lock spray interface and generating a reference ion at 556.2771 Da ([M + H]^+^) or 554.2615Da ([M-H]^-^). The lock spray scan time was set to 0.5 s, with an interval of 15 s, and the acquired data was averaged over three scans. RPLC-MS spectra were obtained in positive and negative ion modes, respectively. For HILIC-MS analysis, only positive mode was employed. The system was controlled by the software package Masslynx V4.1.

The raw data were denoised and smoothed, peak extracted, deconvoluted and annotated automatically by XploreMET (Metabo-Profile Biotechnology (Shanghai) Co., Ltd.) Normalized processed data were subjected to multidimensional and one-dimensional statistical analyses using SIMCA.

### Network Pharmacology Analysis Verification

The pathways with statistically significant differences between the model groups and treated groups as predicted by Tax4Fun were determined, and the correlation between pathways and intestinal flora was identified. In addition, we identified metabolites that differed between the model and medium dose-treated rats, defined as those with VIP (Variable importance in the projection) > 1 as calculated by the OPLS-DA model. The pathways of the differential metabolites were identified using MetaboAnalyst 4.0 (http://www.metaboanalyst.ca/) and the KEGG database and were then compared with the pathways predicted by Tax4Fun. We chose pathways of interest and uploaded them to Cytoscape to analyse the network.

### Data Analysis

The data are presented as means ± standard error (‾x ± SD). One-way ANOVA was used to measure differences among all six groups. SPSS (version 24.0) was used to perform all the statistical analyses, while GraphPad Prism (version 7.0) was used for graphical presentation. SIMICA (Version 14.1) was used to perform the PCA-X and OPLS-DA analyses.

## Results

### Changes in Faecal Intestinal Flora in Rats With GID

**FIGURE 1:**
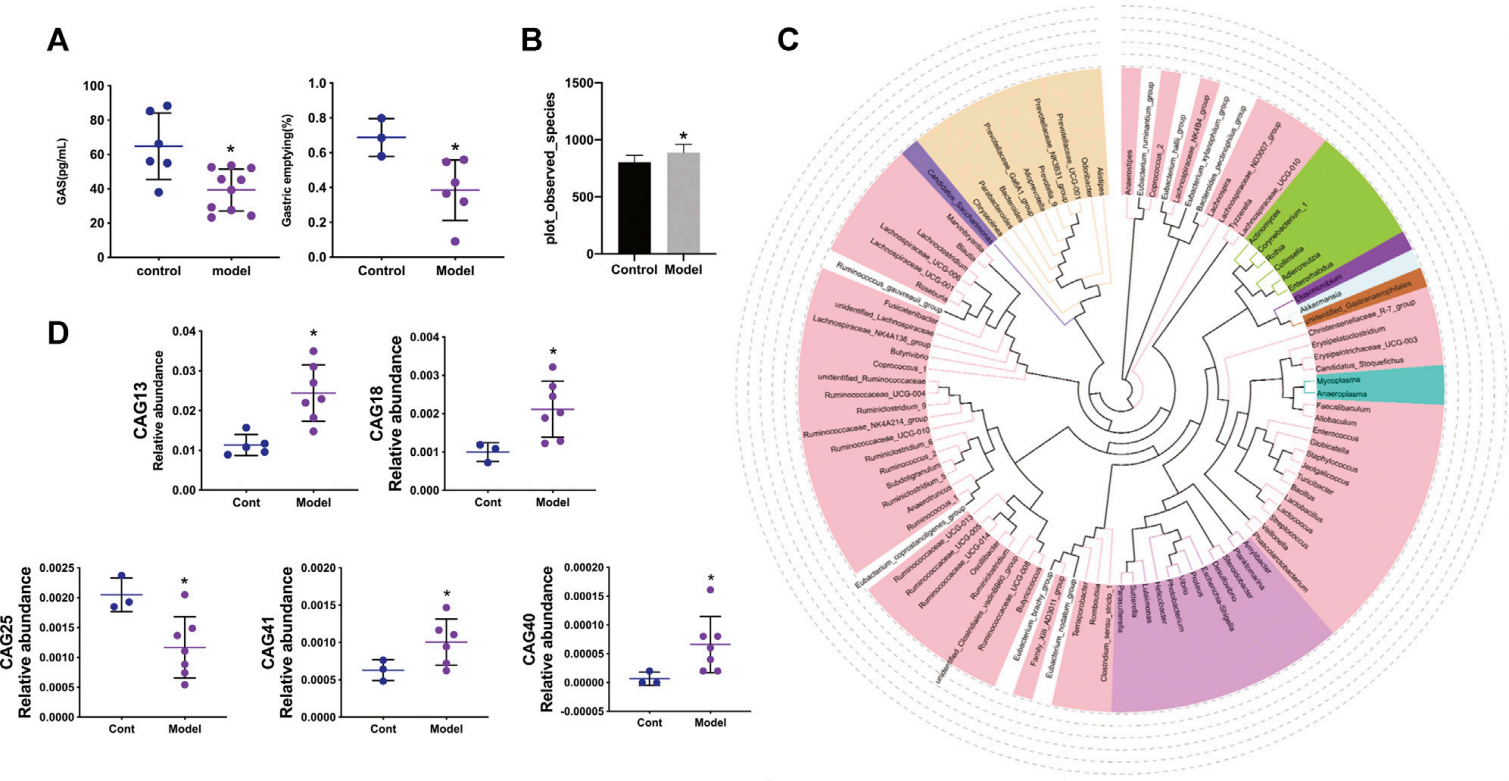
**(A)** The gastric emptying rate between control and model groups and the levels of gastrin in serum between control and model groups **(B)** Species accumulation boxplot and observed species diversity index in control and model groups **(C)**The Phylogenetic tree of the top 100 genus through sequence alignment, different color represents different Phylum **(D)** The relative abundance of CAG13, CAG18, CAG25, CAG40, CAG41 between control and model group. Data are mean ± SD, *p < 0.05 VS the model group.

After the gastric emptying rate and serum level of GAS were evaluated in the GID model, alterations in the intestinal flora were investigated. As shown in Figure 1A, the gastric emptying rate and serum level of GAS were significantly lower in the model group than the control group, indicating that the rat GID model was established successfully.

As shown in Figure 1B, the observed species diversity between the control and model groups was calculated. Compared with the control group, the model group manifested a significant increase in observed species diversity. The phylogenetic tree of the top 100 genera is shown in Figure 1C, in which different colours represent different phyla and the relationship between different phyla can be observed. As shown in Figure 1D, compared with the control group, the relative abundance of CAG in the model group exhibited a significant increase for CAG13 (Erysipelotrichaceae), CAG18 Lachnospiraceae), CAG40 (Streptococcaceae), and CAG41 (Ruminococcaceae), while a significant decrease was observed for CAG25 (Ruminococcaceae).

### Pharmacodynamic Evaluation of XEFP

**FIGURE 2:**
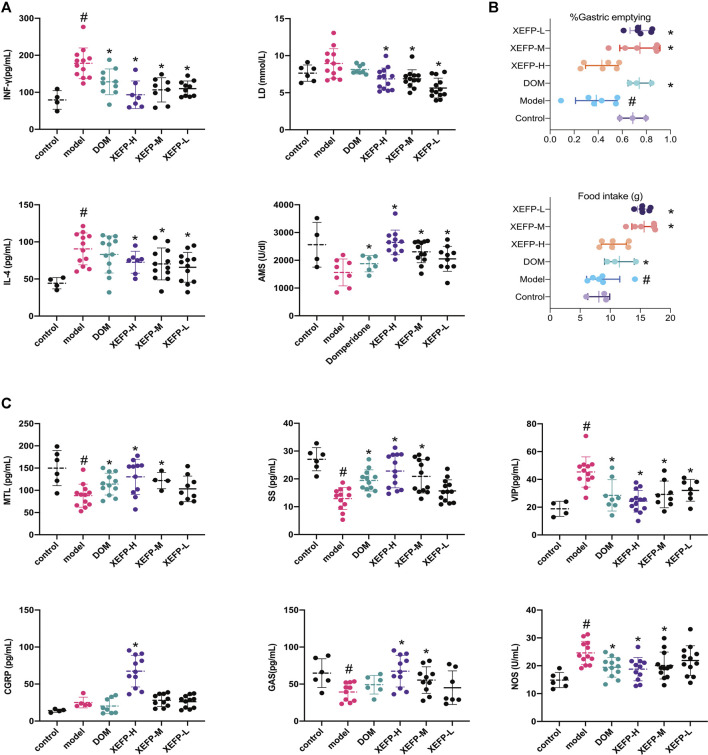
**(A)** XEFP attenuates inflammatory responses of GID rats. The levels of IL-4; The levels of IFN-γ in serum; The levels of AMS in serum; The levels of LD in serum **(B)** XEFP improves the gastric emptying; and the basic data on the food intake of each group **(C)** XEFP modulates the secretion of BGPs of GID rats. The levels of GAS, The levels of MTL, The levels of SS, The levels of CGRP, The levels of NOS, and VIP in serum. The data are presented as the means ± SD. One-way ANOVA followed by LSD post hoc test for multiple comparisons of IL-4, LD and AMS. #*p* < 0.05 VS the control group, *p < 0.05 VS the model group.

To evaluate the pesticide effect of XEFP on GID, the inflammatory factor levels, gastric emptying rate brain gut peptide (BGP) and some blood biochemical factor levels were measured and calculated. As shown in Figure 2A, the inflammatory factors in the model group increased significantly (IL-4 and IFN-γ). In the groups treated with XEFP, however, the factors (IL-4 and IFN-γ) decreased significantly. The AMS levels decreased significantly in the model group compared with the control group, while the treated groups showed a statistically significant trend. Compared with the control group, the LD levels non-significantly increased in the model group, while the levels in the groups that received XEFP significantly decreased compared with the model group.

Here, the gastric emptying rate was used to evaluate whether GID syndrome in rats was improved after the treatment. As shown in Figure 2B, compared with the control group, the model group manifested a significant decrease in the gastric emptying rate. The gastric emptying rate increased in the treated groups compared with the model group.

Figure 2C shows that the BGP levels changed in the model group and treated groups. The GAS, MTL, and SS levels in the model group were significantly decreased compared with those in the control group but were significantly increased in the groups treated with XEFP compared with those in the model group. In addition, the levels of these three indexes in the XEFP-H group were even higher than those in the DOM group, highlighting XEFP as a potential treatment for GID. Furthermore, a dose-response was found across the three XEFP groups. The CGRP, NOS, and VIP levels increased in the model group compared with the control group, and the differences were statistically significant in some cases. The NOS and CGRP levels significantly decreased in the treated groups compared with the model group.

### XEFP Modulates Intestinal Flora in Rats With GID

**FIGURE 3:**
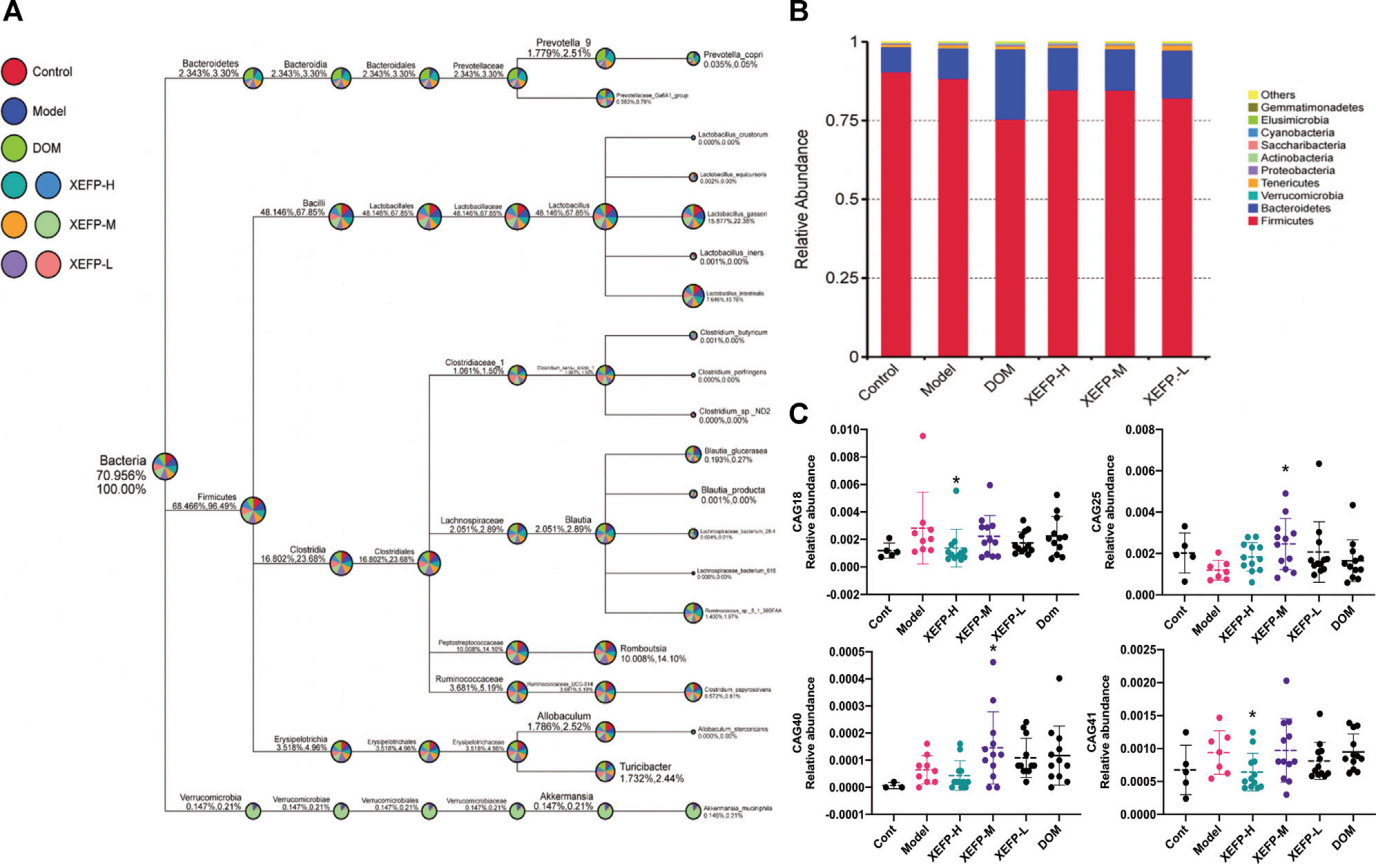
XEFP modulates intestinal flora of GID rats **(A)** The taxonomic tree of all groups **(B)** The bar plot of relative abundance on a phylum level. The size of the sector represents the proportion of the relative abundance of each group; the first number beside the circle represents the percentage of all species and the second represents the percentage of selected species **(C)** The relative abundance of CAG18, CAG25, CAG40, CAG41. The data are presented as the means ± SD. One-way ANOVA followed by LSD post hoc test for multiple comparisons. *p < 0.05 versus the model group.

The intestinal flora in rats with GID was investigated. As shown in Figure 3A, the most common phyla were *Bacteroidetes* and *Firmicutes,* which accounted for 90% of the relative phylum abundance. Figure 3B shows that the intestinal flora changed in the treated groups compared with the model group. The relative abundance of *Firmicutes* in the treated groups increased compared with that in the model group, while the relative abundance of *Bacteroidetes* decreased. The ratio of phyla Firmicutes/Bacteroidetes (F/B) rats decreased in the rats treated with XEFP.

**FIGURE 4:**
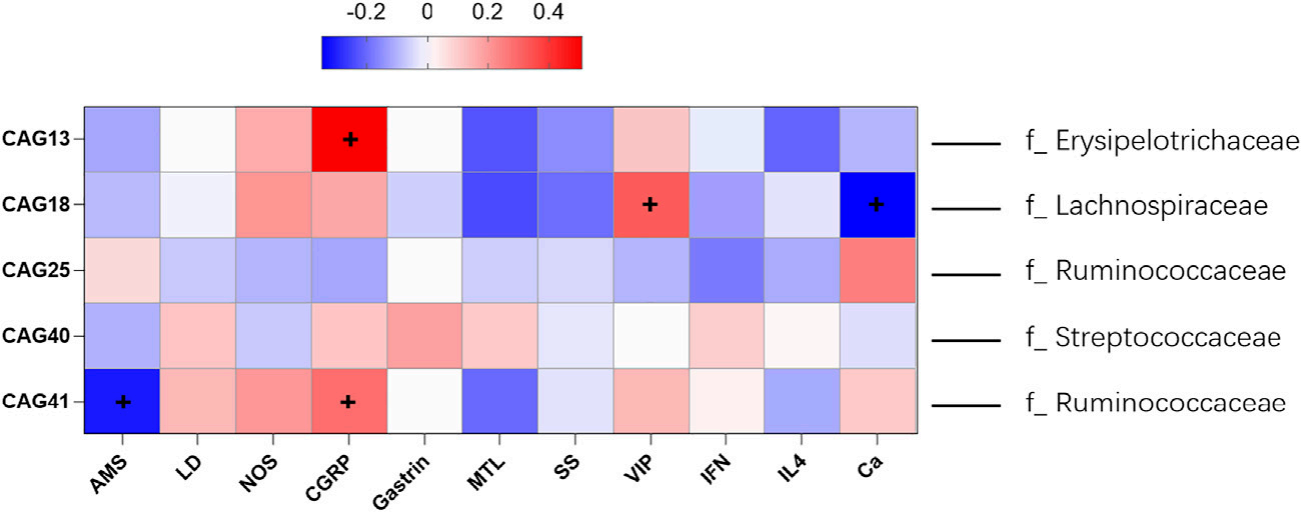
Spearman correlations of five differential CAG and the microbes of highest relative abundance in each CAG with BGPs, inflammatory factors and others. The ‘+’ represent the correlation was statistically significant. The information provided right side represent the dominant family (family with the highest relative abundance).

As shown in Figure 3C, significant decreases in the CAG18 and CAG41 levels were observed in the treated groups compared with the model group, while the CAG18 and CAG41 levels were significantly increased in the model group compared with the control group. A similar phenomenon was observed for CAG25, which showed a significant decrease in the model group compared with the control group and a significant increase in the treated groups compared with the model group. The reduction in CAG18, CAG41, CAG25, and CAG30 indicated that XEFP can relieve GID by altering the microbiota. In addition, the CAG relative abundance index data showing significant and physiological changes were imported into SPSS to perform a Spearman double-tail correlation analysis. *lachnospiraceae* in CAG18 exhibited a significant positive correlation with the level of VIP and a significant negative correlation with the level of CGRP in serum. Ruminococcaceae in CAG 41 exhibited a significant positive correlation with the level of CGRP and a significant negative correlation with the level of AMS (Figure 4).

The functional profiles (KEGG) of the microbial communities were predicted using the Tax4Fun R package. The pathway statistics were selected if they exhibited a significant change in the treated groups compared with the model group. In total, 37 pathways were chosen, which were mainly related to metabolism. Specifically, of 14 metabolism-related pathways, three pathways were related to carbohydrate metabolism, two pathways were related to amino acid metabolism, two pathways were related to energy metabolism and the others were related to the metabolism of terpenoids and polyketides, glycan biosynthesis and lipid metabolism, xenobiotic biodegradation and metabolism, metabolism of cofactors and vitamins and metabolism of terpenoids and polyketides. Interestingly, all three pathways related to carbohydrate metabolism were downregulated in the treated groups compared with the model group. Meanwhile, pathways related to organismal systems (7 pathways) were mainly upregulated in treated groups compared with the model group, including *antigen processing and presentation*, *haematopoietic cell lineage, phototransduction fly*, *progesterone-mediated oocyte maturation*, and *protein digestion and absorption*.

### Verification by Metabolomics Analysis

**FIGURE 5:**
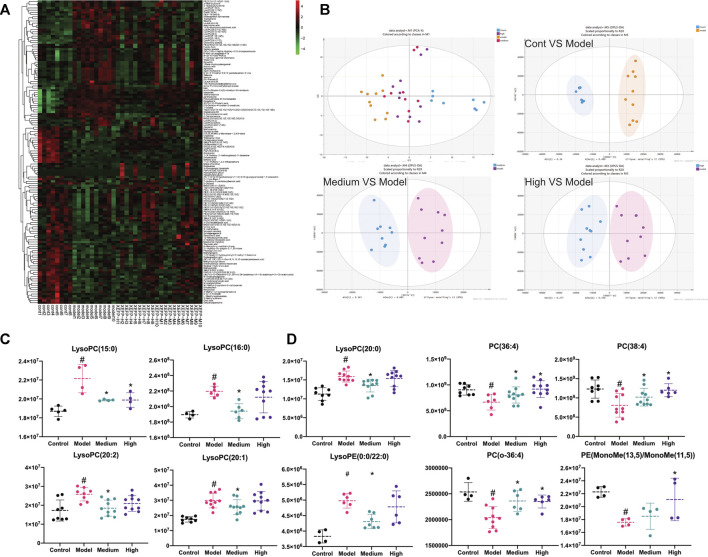
**(A)** Cluster heatmap of all microbiota detected in positive ion mode; **(B)** The PCA-X analysis of control, model, high and medium groups in positive ion mode; OPLS-DA analysis of control VS model in positive ion mode; OPLS-DA analysis of medium VS model in positive ion mode; OPLS-DA analysis of high VS model in positive ion mode. The high group represents XEFP-H group while the medium group means XEFP-M group **(C)** Serum level of LysoPC(15:0), LysoPC(16:0), LysoPC(20:0) LysoPC(20:2 (11Z, 14Z)), LysoPC(20:1 (11Z)), LysoPE (0:0/20:0) changed in model and treated group. Medium group means XEFP-M while High means XEFP-H **(D)** Serum level of PC(16:1 (9Z)/20:3 (8Z,11Z, 14Z), PC(18:2 (9Z, 12Z)/20:2 (11Z, 14Z)), PC(o-16:0/20:4 (8Z,11Z,14Z, 17Z)), PE (MonoMe(13,5)/MonoMe(11,5)) changed in model and treated group. Medium group means XEFP-M while High means XEFP-H. The data are presented as the means ± SD. One-way ANOVA followed by LSD post hoc test for multiple comparisons. *p < 0.05 versus the model group. #*p* < 0.05 versus the control group.

We next performed metabolomics analyses. The cluster heatmap in Figure 5A shows that the model groups had obvious differences compared with the control groups in all the microbiota. Changes also occurred in the treated groups compared with the model groups. As shown in Figure 5B, data after normalization were analyzed by SIMCA using the PCA-X and OPLS-DA models to filter potential metabolites that differed between the model group and treated groups. As shown in Figure 5B, the control group, model group and treated groups could be distinguished easily. Based on the OPLS-DA model, the control group vs model group, model group vs high group, and model group vs medium group were each clearly distinguishable from each other based on serum metabolites.

Differential metabolites whose VIP value > 1 according to the model vs. control OPLS-DA model, model vs medium OPLS-DA model and model vs high OPLS-DA model were chosen and analysed. Most of the metabolites were glyceryl phosphatides (70.8%), including lysophosphatidylcholine (LysoPC), lysophosphatidylethanolamine (LysoPE), phosphatidylcholine (PC) and phosphatidylethanolamine (PE). As shown in Figure 5C, compared with the control group, the LysoPC(LysoPC(16:0)), LysoPC(20:2 (11Z, 14Z)), LysoPC(15:0), LysoPC(20:2 (11Z, 14Z)), LysoPC(20:1 (11Z)), LysoPC(15:0), LysoPC(20:0) and LysoPE (LysoPE (0:0/20:0)) levels were significantly increased in the model group; these levels were also increased significantly in the treated groups compared with the model group. In addition, as shown in Figure 5D, the PC(PC(16:1 (9Z)/20:3 (8Z,11Z, 14Z)), PC(18:2 (9Z, 12Z)/20:2 (11Z, 14Z)), PC(o-16:0/20:4 (8Z,11Z,14Z, 17Z))) and PE (PE (MonoMe(13,5)/MonoMe(11,5))) levels were decreased significantly in the model group compared with those in the control group; these levels in the treated groups were increased significantly compared with those in the model group.

### Network Analysis Verification

**FIGURE 6:**
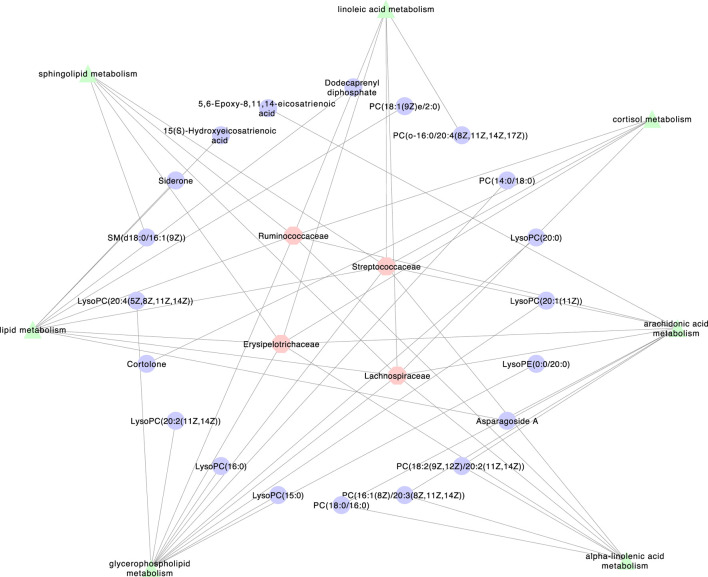
The network among Intestinal flora, pathways and metabolite.

As shown in Figure 6, a network was created among the intestinal flora, pathways and metabolites to explain how XEFP affects GID at the microbiota level. Four main microbiotas with significantly changed CAGs, Lachnospiraceae, Ruminococcaceae, Streptococcaceae and Erysipelotrichaceae, altered 20 main metabolites through seven pathways comprising *arachidonic acid metabolism, glycerophospholipid metabolism, linoleic acid metabolism, alpha-linolenic acid metabolism, lipid metabolism, sphingolipid metabolism* and *cortisol metabolism*.

## Discussion

The composition of the intestinal flora differentiates people with different physiological and pathological states, which may result in CNS and ENS alterations (Fan and Pedersen, 2021). A growing number of studies have shown that the intestinal flora stimulates physiological functions in many ways, including energy metabolism, nutrient metabolism, and immunoreaction (Michaudel and Sokol, 2020). Patients with functional gastrointestinal disorder always consume a particular kind of food, which implies that the microbiota in the patient's alimentary system changes in some ways (Sayuk and Gyawali, 2020). In our study, we used a microbiome and metabonomics integration strategy to reveal alterations in the intestinal flora and metabolites in patients with GID. In addition, we examined XEFP, a TCM used to treat patients with GID, and found that it can reverse the imbalance in the intestinal flora and revert some of the metabolite increases and or decreases in rats with GID.

It has been reported that structural alterations of the intestinal flora modulate physiological function and even the pathological state of the host (Caenepeel et al., 2020). In recent years, an increasing number of studies have investigated whether the intestinal flora plays a regulatory role in GID. Five CAGs changed significantly in faecal in rats with GID compared with the control group, among which Ruminococcaceae is one of the human butyrate-producing gut bacterial strains. The change in the relative abundance of Ruminococcaceae may yield a modification of short chain fat acids (SCFAs), which is also a factor in host homeostasis (Rastogi et al., 2020; Soto-Martin et al., 2020). Streptococcaceae (Kok and Hutkins, 2018; Fukui et al., 2020), whose relative abundance in upper GI increased, is considered fasting the process of metabolic disorders and diabetes (Abdulamir et al., 2011). Although tested in faecal sample, the relative abundance of *Streptococcacea* increased in the GID group, while decreased in GID rats treated with XEFP. At the same time, the similar phenomenon occurred on relative abundance of obesity-associated Erysipelotrichaceae (Kamp et al., 2021) and diet-induced obesity-related Lachnospiraceae (Zhao et al., 2017), which comprise most of the CAGs. In the analysis of the α diversity of the intestinal flora in faecal, a change in the ratio of Firmicutes to Bacteroidetes phyla between the GID model group and treated groups was found, and the ratio of Firmicutes to Bacteroidetes phyla in upper GI is considered to have a close relationship with FD (Hills et al., 2019). The GID pathological state may lead to an imbalance in the intestinal flora in upper GI, thus influents the balance of intestinal flora in faecal which can alter the SCFAs and subsequently affect GID. It has been reported that the increase in Ruminococcaceae and decrease in Streptococcaceae*,* Erysipelotrichaceae Lachnospiraceae in upper GI is a predictor of a good prognosis for patients. Identically, XEFP-induced increase in bacteria Ruminococcaceae and decrease in Streptococcaceae*,* Erysipelotrichaceae and Lachnospiraceae in intestinal flora of faecal might be an evidence for XEFP to relief GID and improve metabolic disorders.

Accumulating evidence shows that some BGPS and blood chemical factors have numerous ties with GID. In recent years, an increasing number of studies have focused on the brain-gut axis and the BGPs represented by GAS, MTL, VIP, SS and NOS. It has been reported that GAS has a positive correlation with GID treatment in that it can increase gastric vitality, and the same function has also been attributed to MTL (Deloose et al., 2019). VIP is an important chemical released by the ENS and comes into effect in the gastrointestinal area by producing NO and relaxing the smooth muscle in the gastrointestinal tract (Lai et al., 2017). SS analogues have been used as a treatment for digestive diseases (Gomes-Porras et al., 2020). Compared with positive drug, XEFP did have some advantages in reversing the imbalances in microbiota in some extent. As shown in Figure 3C, the imbalances of CAGs reversed in high dose and the medium dose of XEFP while in DOM group, there was no change in CAGs, compared with the model group. According to our research, XEFP can relieve GID by modulating BGPs by decreasing the serum levels of VIP and NOS while increasing the levels of MTL, SS, and GAS. In the Spearman double-tail correlation analysis we performed, the Lachnospiraceae in CAG18 had a significant positive correlation with the level of VIP and a significant negative correlation with the level of CGRP in serum. The Ruminococcaceae in CAG 41 had a significant positive correlation with the level of CGRP and a significant negative correlation with the level of AMS. These results indicate that XEFP is likely to regulate BGPs by restoring the balance of intestinal flora in faecal.

The intestinal flora affects host physiology by producing thousands of metabolites. The crosstalk between the host and microorganisms exerts an influence by altering signalling pathways and metabolic reactions (Krautkramer et al., 2020; Lin et al., 2020). To better understand this interaction, we studied the change in metabolites. Most of the metabolites that changed significantly in the groups treated with XEFP were glyceryl phosphatides (70.8%). It has been reported that the LysoPC levels in serum are a feasible candidate marker for patients with gastrointestinal disease, while it associated with ≥5% weight loss through *glycerophospholipid metabolism* (Miller et al., 2019), which was also detected changed in our study. However, most of the lysophosphatidylcholines (LysoPC and LysoPE) were increased in the model groups, while most of the phosphatidylcholines (PC and PE) were decreased in the model groups. It can be deduced that an imbalance of the intestinal flora also leads to an imbalance between lysophosphatidylcholine and phosphatidylcholine. The traditional TCM XEFP can correct these imbalances. In addition, significant changes were observed in metabolites participating in several metabolic pathways, such as *arachidonic acid metabolism*, *glycerophospholipid metabolism, linoleic acid metabolism, alpha-linolenic acid metabolism, sphingolipid metabolism* and *ether lipid metabolism*. Interestingly, in Tax4Fun pathway analysis of the intestinal flora, *ether lipid metabolism* was also significantly changed. It can be deduced that XEFP can make alteration to the relative abundance of some key intestinal flora including Ruminococcaceae*,* Streptococcaceae, Erysipelotrichaceae and Lachnospiraceae by the *ether lipid metabolism* pathway then relief the symptom of GID. The imbalance of lysophosphatidylcholines inverted after treated with XEFP confirmed the curative effect of XEFP on GID.

In conclusion, an integrative microbiome and metabonomics strategy was used to evaluate alterations of the intestinal flora and metabolites in rats with GID to identify an imbalance in the GID pathological state. XEFP can reverse these imbalances in rats with GID. It can be concluded that 1) disharmony of the intestinal flora in faecal occurs in rats with GID; 2) disorders in the intestinal flora of faecal in GID pathological state, including Lachnospiraceae, Ruminococcaceae, Streptococcaceae and Erysipelotrichaceae, coincides with the imbalance in metabolites, including glyceryl phosphatides; and (3) XEFP can relive GID and improve the disorders of metabolic and faecal intestinal flora. This study represents a comprehensive survey of the interaction between GID and the intestinal flora and a systematic evaluation of modulation by a multicomponent drug. In addition, this is the first study to investigate the mechanism by which XEFP affects GID on multiple levels.

## Figures and Tables

**FIGURE A1:**
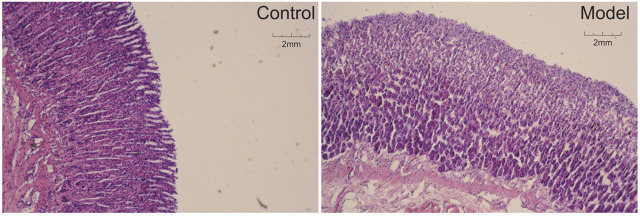
Hematoxylin and eosin (HE) staining for gastric morphology (photomicrography, × 4).

## Data Availability

The datasets presented in this study can be found in online repositories. The names of the repository/repositories and accession number(s) can be found below: https://www.ncbi.nlm.nih.gov/, PRJNA719295.

## Appendix

### Evaluation of Pathological Sections of Gastric Tissues

After anesthetizing with 1% pentobarbital sodium, the stomachs of rats were removed, cut open. And immersed in 4% formalin for 72 h at 4°C. Sections from paraffin-embedded tissue specimens were processed for HE staining for histologic evaluation (Figure A1).

## Glossary

AMS: amylase
BGP: brain gut peptide
CNS: central nervous system
CGRP: calcitonin gene-related protein
DOM: domperidone
ESI: electrospray ionization
FD: functional dyspepsia
GP: gastroparesis
GID: gastrointestinal disorder
GAS: astrin
HILIC: hydrophilic interaction chromatography
IAA: Iodoacetamide
IL-4: perinterleukin 4
IFN-γ: interferon-γ
LD: lactic acid
LysoPC: lysophosphatidylcholine
LysoPE: phosphoethanolamine
MTL: Motilin
NOS: nitric oxide synthase
OTUs: operational taxonomic units
PC: phosphatidylcholine
PE: phosphoethanolamine.
Q-TOF: quadrupole-time-of-light
RPLC: reversed-phase liquid chromatography
SS: somatostatin
SCFAs: short chain fat acids
SDS-PAGE: SDS-polyacrylamide gel electrophoresis
TCM: traditional chinese medicine
VIP: vasoactive intestinal peptide
XEFP: xiaoerfupi granule.
